# Reviewer Acknowledgment

**DOI:** 10.1002/kjm2.12620

**Published:** 2022-11-05

Consistent high‐quality of papers published in “Kaohsiung Journal of Medical Sciences” can only be maintained with the cooperation and dedication of a number of expert referees. The Editors would like to thank all those who have donated the hours necessary to review, evaluate and comment on manuscripts; their conscientious efforts have enabled the journal to maintain its tradition of excellence. We are grateful to the following reviewers for their contributions during 2022.

A

Amrousy Doaa El

B

Bair Ming‐Jong

C

Chan Te‐Fu

Chang Hsueh‐Wei

Chang Je‐Ken

Chang Jer‐Ming

Chang Kuo‐Wei

Chang Kwang‐Yu

Chang Long‐Sen

Chang Ting‐Ting

Chang Wen‐Tsan

Chang Wun‐Shaing

Chao Sheau‐Chiou

Chen Chieh‐Chang

Chen Chien‐Hung

Chen Chien‐Lin

Chen Ching‐Chow

Chen Chung‐Hwan

Chen Chung‐Ming

Chen Fang‐Ming

Chen Kao‐Ching

Chen Po‐See

Chen Shin‐Cheh

Chen Yen‐Hsu

Chen Yi‐Fu

Chen Yih‐Fung

Chen You‐Yi

Chen Yuh‐Fung

Chen Yuk‐Kwan

Cheng ​Ya‐Jung

Cheng Ching‐Feng

Cheng Wen‐Fang

Chien Chih‐Yen

Chien Rong‐Nan

Chiou Tzeon‐Jye

Chiu Ching‐Feng

Chou Cheng‐Yang

Chou Wen‐Chin

Chu Che‐Sheng

Chuang Cheng‐Keng

Chuang Kai‐Jen

Chuang Shuang‐En

F

Fang I‐Mo

Fang Sheen‐Yie

Fu Wen‐Mei

G

Gurzu Simona

H

Han Xue‐Chang

He Jing

Ho Hong‐Nerng

Hong Yi‐Ren

Hsiao Geor‐Ge

Hsiao Hui‐Hua

Hsieh Sen‐Yung

Hsu Chung‐Yao

Hsu Jong‐Hau

Hsu Pei‐Ling

Hsu Po‐Chao

Hu Chu‐Sung

Huang Chung‐Feng

Huang Huei‐Sheng

Huang Jee‐Fu

Huang Shiu‐Feng

Huang Shu‐Pin

Huang Tze‐Sing

Huang Yi‐Hsiang

Huang Yi‐Shuian

Hung Chao‐Hung

Hung Chih‐Hsing

Hung Jan‐Jong

Hung Wen‐Chun

Hwang Tsann‐Long

J

Jee Shiou‐Hwa

Juan Yung‐Shun

Juang Jyh‐Ming

K

Kuo Cheng‐Deng

Kuo Kung‐Kai

L

Lai Jenn‐Haung

Lai Yu‐Hung

Lan Cheng‐Che

Lee Chieh‐Ming

Lee Chien‐Hsing

Lee Hsiang‐Chun

Lee Hsiang‐Ying

Lee I‐Hui

Lee Kuo‐Ting

Lee Simon

Lee Su‐Shin

Lee Yen‐Mei

Lee Yi‐Chen

Li Chia‐Yang

Lin Chiao‐Yun

Lin Chien‐Chung

Lin Chii‐Jeng

Lin Han‐Chieh

Lin Hsuan‐Hwai

Lin Hui‐Ju

Lin Jiunn‐Lee

Lin Shi‐Ming

Lin Teng‐Nan

Lin Wan‐Wan

Lin Yi‐Tsung

Lin Zu‐Yau

Liu Chen‐Hua

Liu Chun‐Jen

Liu Li‐Feng

Liu Ping‐Yen

Liu Yen‐Chin

Liu Yi‐Wen

Liu Yo‐Tsen

Liu Yu‐Ping

Lo Gin‐Ho

Lou Pei‐Jen

Lu Ching‐Liang

Lu I‐Cheng

M

Ma Hsu

P

Peng Cheng‐Yuan

S

Sheen‐Chen Shry‐Ming

Sheu Shwu‐Jiuan

Shiah Shine‐Gwo

Shieh Sheau‐Yann

Shyue Song‐Kun

Su Chun‐Li

Su Ming‐Yao

Su Yu‐Chieh

Suen Jau‐Ling

Sun Wei‐Zen

Sung Chih‐Chien

Sung Junne‐Ming

T

Tai Ming‐Hong

Tsai Eing‐Mei

Tsai Kuo‐Wang

Tsai Rong‐Kung

Tsai Wei‐Chung

Tseng Yau‐Lin

Tu Hung‐Pin

Tzeng Yuan‐Sheng

W

Wang Cheng‐Ping

Wang Jaw‐Yuan

Wang Jing‐Houng

Wang Shen‐Nien

Wang Shyu‐Jye

Wu Bin‐Nan

Wu Chew‐Wun

Wu Chin‐Chen

Wu Deng‐Chyang

Wu I‐Chen

Wu June‐Hsieh

Wu Ruey‐Meei

Wu Yih‐Ru

Y

Yang Chih‐Jen

Yang Chuen‐Mao

Yang Kai‐Chien

Yang Shei‐Dei

Yang Yen‐Kuang

Yang Yung‐Li

Yeh Jwu‐Lai

Yeh Kuang‐Hui

Yeh Ming‐Lun

Yeh Ta‐Sen

Yen Cheng‐Fang

Yen Hsueh‐Wei

Yu Chong‐Jen

Yu Dah‐Shyong

Yu Hong‐Ren

Yuan Shyng‐Shiou

Z

Zhang Jing‐Ru

Zhang Yu‐Tao

